# Responsiveness and minimal important change for the quick-DASH in patients with shoulder disorders

**DOI:** 10.1186/s12955-018-1052-2

**Published:** 2018-12-10

**Authors:** Cecilie Rud Budtz, Johan Hviid Andersen, Nils-Bo de Vos Andersen, David Høyrup Christiansen

**Affiliations:** 1 Department of Occupational Medicine, Regional Hospital West Jutland, University Research Clinic, Gl. Landevej 61, 7400 Herning, Denmark; 2 Central Denmark Region, Primary Health Care and Quality Improvement, Skottenborg 26, 8800 Viborg, Denmark; 3 Department of Clinical Medicine, Health, Aarhus University, Palle Juul-Jensens Boulevard 82, 8200 Aarhus, Denmark

**Keywords:** Outcome measure, Responsiveness, Quick-DASH

## Abstract

**Background:**

Responsiveness and minimal important change (MIC) are central measurement properties when interpreting scores from health questionnaires. The aim of the study was to evaluate the responsiveness and MIC of the Danish version of the shortened version the Disabilities of the Arm, Shoulder and Hand questionnaire (Quick-DASH) in patients with shoulder disorders referred to primary care physiotherapy treatment.

**Methods:**

The study included 261 patients who completed questionnaires at baseline and 3 and 6 months follow up. Absolute and relative change scores was analysed using receiver-operating-characteristics (ROC) curve analysis with the Patient Global Impression of Change (PGIC) as external anchor.

**Results:**

At both 3 and 6 months follow up, the Area under the Curve (ROC AUC) exceeded 0.70 and MIC was 9.1 and 13.6 at 3 and 6 months respectively.

**Conclusion:**

The Danish version of the Quick-DASH demonstrated adequate ability to measure changes in disability over 3 and 6 months in patients with shoulder disorders undergoing primary care physiotherapy treatment.

## Background

Reliable, valid and responsive outcome measures are important when evaluating the effect of treatment. The Disabilities of the Arm, Shoulder and Hand questionnaire (DASH) is a 30 item questionnaire designed to measure symptoms and physical functioning in patients with any or multiple musculoskeletal symptoms of the upper extremity [1]. A major advantage of this questionnaire is that it can be used for any upper extremity evaluation meaning versatility for clinicians and researchers [2]. The DASH has previously been cross cultural adapted to Danish and has been found reliable, valid and responsive among orthopaedic patients with various hand and shoulder diagnoses [3, 4]. However, it is important that a questionnaire is short and easy to complete thereby minimizing the burden on the respondent and limiting missing data. Accordingly, a shortened version of the DASH has been developed (Quick-DASH) [2]. Although such short form questionnaires seem as an attractive and sensible choice, it is essential to insure that measurement properties are maintained [2]. This also applies to the Danish version of the Quick-DASH, which only recently has been evaluated with respect to reliability and construct validity in patients with wrist fractures [5] or total wrist arthroplasty [6].

A central measurement property of scales to assess treatment outcomes is responsiveness. Responsiveness is defined as the ability of a questionnaire to detect change over time in the domain of interest, and it includes the Minimal Important Change (MIC) which is the smallest change in score that would likely be important from the patient’s perspective [7]. To ensure the MIC can be distinguished from measurement error, the MIC should ideally exceed the Minimal Detectable Change (MDC) representing the smallest within-person change in score that can be interpreted as real change beyond measurement error. Responsiveness has been evaluated for Quick-DASH in other countries, various populations and settings with divergent results [8, 9]. Responsiveness of the Danish version of the Quick-DASH has however never been evaluated using the recommended anchor-based method or in a population of shoulder patients. As responsiveness and MIC are likely to depend on population and contextual characteristics, evaluation should be conducted within the setting in which the questionnaire is going to be utilised [10]. The aim of the present study was to evaluate responsiveness and MIC of the Danish Quick-DASH in patients with shoulder disorders referred to primary care physiotherapy treatment, thereby adding to earlier findings on the use of this questionnaire. We hypothesised that the Quick-DASH would 1) demonstrate adequate ability to discriminate between improved and unchanged patients and 2) MIC thresholds would exceed the minimal detectable change.

## Methods

### Design and population

The study was nested in a larger prospective cohort study of patients seeking treatment for neck, shoulder, or low-back pain in 23 physiotherapy practices across Denmark from January to June 2016 [11]. Consecutive patients were invited to participate if they were aged 18 years or above and able to understand Danish well enough to self-complete the questionnaires. For the current study patients presenting with shoulder pain were included.

### Data collection

Questionnaire and clinical data were collected using an existing web-based clinical database (www.fysdb.dk). Patients who agreed to participate in the study were asked to complete online questionnaires 1–2 days prior to the first physiotherapy consultation (baseline) and at 3 and 6 months follow up. Participants were notified by e-mail when the follow-up questionnaires were available for completion. The questionnaires included information on gender, age, education level, pain (scale 0–10) and disability (Quick-DASH).

### Quick-DASH and patient global impression of change scale

The Quick-DASH questionnaire contains 11 items (scored on a 0–5 Likert scale) that measure upper limb physical disability and symptoms. Each item has 5 response options from 1 (no difficulty to perform, no symptom or no impact) to 5 (unable to do, very severe symptom or high impact). The responses are summed to a raw score and converted to a 0 (no disability) to 100 (most severe disability) score using the following formula [(sum of score/*n*)-1] × 25, *n* being the number of completed responses [2].

The Patient Global Impression of Change (PGIC) at 3 months formed the external anchor. Patients were asked to rank their overall state in relation to time of referral on a 7 point scale from much better to much worse. The PGIC scale is widely used in clinical research as a way to quantify the patient’s perception of improvement over time [12].

### Measurement error of the quick-DASH

The measurement error of the Quick-DASH has never been established in a population of Danish primary care patients with shoulder disorders. Reliability was consequently established based in a subsidiary sample of 30 consecutive shoulder patients (mean (SD) age 49 [12]; 18 women)). The patients filled in the Quick-DASH twice with a median of 5 days [IQR 3;7] between administrations. The intraclass correlation coefficient (ICC) was 0.87 (95% CI: 0.79; 0.96)) and the Standard Error of Measurement (SEM) was estimated to 6.0 (95% CI: 4.7; 8.0), which yielded a Minimal Detectable Change (MDC) of 16.5 (95% CI 13.1; 22.2) [10].

## Statistical analyses

Descriptive statistics were calculated and presented. Responsiveness was evaluated using an external anchor approach as recommended in the COSMIN guidelines [3]. Receiver-operating-characteristics (ROC) curve analysis of the absolute and relative change scores in the Quick-DASH was used to assess the measurements ability to correctly classify patients as improved (much better or better) or unchanged (little better, unchanged or little worse) according to the PGIC scale. As only few patients (*n* = 3 at 3 months, *n* = 5 at 6 months) were worse or much worse, responsiveness to worsening was not analysed. In ROC curve analysis, sensitivity and 1-specificity are plotted at several cut-off points, and the area under the curve (ROC AUC) can be estimated. An ROC AUC greater than 0.70 is considered adequate [10]. All absolute and relative changes and ROC AUC were calculated with 95% CI. Minimal important change (MIC), estimated using the cut off point at the ROC curve closest to the upper left-hand corner and where sensitivity and specificity was most balanced, represents the score that best discriminates between improved and unchanged patients (ROC MIC). Furthermore, we calculated the 95% limit cut off point (MIC 95% upper limit), defined as the upper limit of the distribution of patients who were not importantly changed according to the external anchor (mean change + 1.645 x SD change of the unchanged group) [13]. Missing items in the Quick –DASH were handled according to recommendations meaning if only 1 item was missing, the score was divided by 10 instead of 11 and if more than 1 item were missing the total score was not calculated [2]. All statistical analyses were performed using STATA Version 15 (StataCorp LP, College Station, TX).

## Results

A total of 341 patients with shoulder conditions were referred from general practitioner to physiotherapy treatment from January to June 2016 of which 5 were excluded (age under 18 (*n* = 4) and suspicion on serious disease (*n* = 1)). A total of 336 patients were invited to participate in the study, 30 patient declined to participate and 45 patients had more than 1 item missing in their baseline Quick-DASH questionnaire, resulting in 261 patients included at baseline (78%). A total of 73 patients at 3 months and 85 patients at 6 months were lost to follow up, resulting in 188 patients (72%) at 3 months and 176 patients (67%) at 6 months to be included in the analyses. Baseline characteristics of included patients (*n* = 261) are presented in Table 1.

**Table 1:** Baseline characteristics (*n* = 261)

Variable	Value*
Sex	
Female	139 (53)
Male	122 (47)
Age, mean (SD)	52 (14)
Occupational status	
Employed	179 (69)
Unemployed	6 (2)
Retired/early retirement/flex job/disability pension	58 (22)
Student/on leave	14 (5)
Missing	4 (2)
Sick leave because of shoulder symptoms	24 (9)
QuickDASH 0–100, mean (SD)	35.1 (18.4)
Pain 0–10, mean (SD)	5.7 (2.1)

Abbreviations: *SD* standard deviation, Quick-DASH, shortened version of the Disabilities of the Arm, Hand and Shoulder

*Values are n (%) unless stated otherwise

No differences in baseline variables between responders and non-responders at 3 and 6 months were detected (data not shown).

**Fig. 1:**
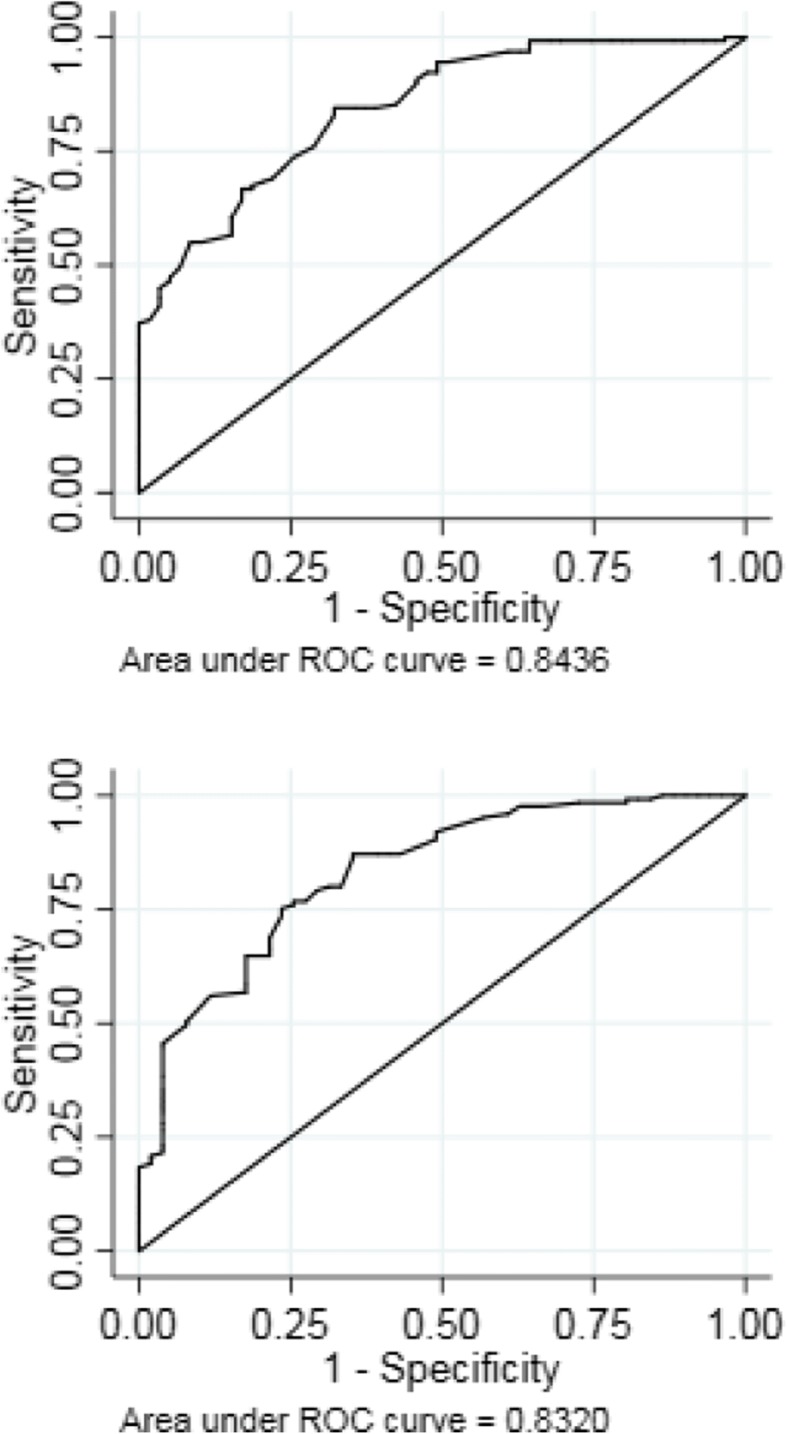
Receiver-Operating-Characteristics (ROC) curve for the Quick-DASH at 3 months (upper) and 6 months (lower) follow up. The point nearest the upper left hand corner represents the minimal important change (MIC ROC)

At 3 and 6 months a total of 129 and 125 patients were improved and 59 and 51 were unchanged respectively. Table 2 present mean and relative change scores, ROC curve statistics and ROC MIC values at 3 and 6 months. The absolute ROC AUC was 0.84 (0.79;0.90) at 3 months and 0.83 (0.77;0.90) at 6 months. At 3 months the ROC MIC was 9.1 points (sensitivity: 73.6%; specificity: 74.5%; correctly classified: 73.9%) and at 6 months the ROC MIC was 13.6 points (sensitivity: 75.2%; specificity: 76.5%; correctly classified: 75.6%). Figure 1 presents the ROC curve at 3 and 6 months.

**Table 2:** Mean change scores, ROC curve statistics and MIC values for the Quick-DASH at 3 and 6 months*

	3 months	6 months
Absolute mean changes		
Improved	21.4 (18.4;24.3)	24.9 (21.8;28.0)
Unchanged	1.8 (−1.27;4.91)	4.9 (1.0;8.8)
Relative mean changes, %		
Improved	58.1 (52.3;63.8)	68.7 (63.8;73.7)
Unchanged	−3.3 (−19.1;12.6)	6.8 (− 8.8;22.3)
Absolute ROC AUC	0.84 (0.79;0.90)	0.83 (0.77;0.90)
Relative ROC AUC	0.86 (0.81;0.92)	0.90 (0.85;0.95)
MIC values		
MIC ROC ^a^	9.1	13.6
MIC 95% upper limit ^b^	21.3	27.8
MIC percentages ^c^	33.3	41.7

Abbreviations: *AUC* Area Under the Curve, *MIC* Minimal important change, *ROC* Receiver operating characteristics, Quick-DASH, shortened version of the Disabilities of the Arm, Hand and Shoulder

*Values in parentheses are 95% confidence interval. 3 months: improved (*n* = 129); unchanged (*n* = 59), 6 months: improved (*n* = 125); unchanged (*n* = 51)

^a^ Estimated as the optimal cutoff point of the ROC curve using absolute change scores

^b^ Estimated as the 95% cutoff limit for the unchanged group

^c^ Estimated as the optimal cutoff point of the ROC curve using relative change scores

## Discussion

The Danish version of the Quick-DASH demonstrated adequate responsiveness among physiotherapy shoulder patients treated in primary care. At both 3 and 6 months follow up, the lower limit of the 95% CI of ROC AUC estimates exceeded 0.70, suggesting that the questionnaires ability to correctly classify patients as improved or unchanged was adequate. The estimated ROC MIC at 3 and 6 months was 9.1 and 13.6 points respectably and thereby did not exceed the Minimal Detectable Change (MDC) threshold of 16.5 points established for the present study.

The large cohort of consecutive patients with shoulder disorders are likely to be representative of shoulder patients seen in primary care physiotherapy. The patients had relatively high pain scores and modest disability scores at baseline, which is similar to findings from other Danish cohorts of primary care musculoskeletal physiotherapy patients [14, 15]. A limitation of the study was the participation rate at baseline (78%) and modest follow-up rates at 3 and 6 months (72 and 67% respectively). This may have affected the generalizability of our findings and we cannot preclude attrition bias, although no differences in baseline variables between responders and non-responders at 3 and 6 months were detected. Also 45 patients were excluded at baseline as they had more than one item missing in the QuickDASH, indicating the questionnaire may have problems with content validity. Furthermore, the use of transitional scales, such as the PGIC scale, as external anchor has been questioned, as it may be subject to recall bias [7, 16]. However, it remains the only option as no gold standard exists for measuring subjective changes in disability over time [12, 16]. The test-retest reliability was based on a separate but similar population and it cannot be precluded that the SEM and MDC would have differed if the test-retest had been performed in a subset of the large cohort. Also the test-retest was performed on 30 patients, which only meets a fair rating in the COSMIN rating of the methodological quality [17]. The results were however in line with previously reported ICC values which was evaluated in 30 and 22 patients [13, 14], and the results are therefore considered reliable.

Only a few studies have examined responsiveness and MIC of the Quick-DASH in patients undergoing physiotherapy treatment for shoulder disorders [17–20]. Our findings on ROC AUC coincides with 3 studies evaluating responsiveness using PGIC as external anchor, with a ROC AUC ranging from 0.82 to 0.86 [17–19]. There is considerable discrepancies in the reported MIC values in these previous studies, as they range from 8 to 19 [17, 18, 20]. These findings are all based on follow-up periods of 4–6 weeks, and to our knowledge no other study has examined MIC over a longer follow-up period in a similar population. Interestingly, our findings indicate that MIC is not stable over time, as it increases from 9.1 to 13.6 points. The MDC of 16.5 points represent the smallest within-person change in score that can be interpreted as real change beyond measurement error. In contrast to previous studies [13, 16] MIC did not exceed the MDC in the present study. However, it should be noted that these previous studies calculated the MDC with a 90% confidence level (MDC^90^) (11–13 points), whereas we calculated the 95% confidence level (MDC^95^). The estimated MDC^90^ in our study would be 13.9 points, and thereby not far from these previous reported [20, 21] results and almost equal to our estimated MIC at 6 months. When interpreting individual absolute change in patients in clinical practise and research the MIC 95% upper limit may be a preferable choice, on the other hand, as MIC estimates are likely to be influenced by baseline scores [22], MIC percentages may be an even better choice. Our findings on relative MIC scores of 33 and 41% at 3 and 6 months coincide with common thresholds of clinically relevant important change of > 30% improvement from baseline to follow-up [21].

In addition, the findings that both absolute and relative minimally important change scores vary over time calls for further investigation into variations over time.

## Conclusion

The Danish version of the Quick-DASH demonstrated adequate ability to measure changes in disability over 3 and 6 months in patients with shoulder disorders undergoing primary care physiotherapy treatment. Minimal important change values in ROC estimates were 9.1 and 13.6 points at 3 and 6 months respectively and did not exceed the MDC_95_ value in the present study. To insure that individual change scores will exceed measurement error, the MIC 95% upper limit of 21 and 28 or the relative change scores of 33% or 41% may be more preferable when interpreting clinical importance of individual changes in Quick-DASH score over time.

## Abbreviations

AUC: Area Under the Curve
CI: Confidence Interval
MDC: Minimal Detectable Change
MIC: Minimal Important Change
PGIC: Patient Global Impression of Change
Quick-DASH: Shortened version of the Disabilities of the Arm, Shoulder and Hand questionnaire
ROC: Receiver-Operating-Characteristics
SD: Standard Deviation
SEM: Standard Error of Measurement
